# Editorial: Bridging electrophysiological markers from preclinical to clinical neuropsychiatric research

**DOI:** 10.3389/fpsyt.2026.2035905

**Published:** 2026-10-30

**Authors:** Hongjian Pu, Miranda F. Koloski

**Affiliations:** 1 Department of Neurology, University of Pittsburgh, Pittsburgh, PA, United States; 2 Geriatric Research, Education and Clinical Center, Veterans Affairs Pittsburgh Healthcare System, Pittsburgh, PA, United States; 3 Department of Psychiatry, University of California, San Diego, San Diego, CA, United States; 4 Mental Health, VA San Diego Healthcare System, San Diego, CA, United States

**Keywords:** deep brain stimulation (DBS), electroencephalography (EEG), electrophysiological markers, event-related potentials (ERPs), functional near-infrared spectroscopy (fNIRS), local field potentials (LFPs), neuropsychiatric disorders (NPD), neurostimulation

Neuropsychiatric disorders reflect complex interactions among neural circuits, cognition, physiology, and the environment. Although clinical diagnoses guide care, people with the same diagnosis can differ substantially in symptoms, underlying biology, and treatment response. Electrophysiological measures provide objective assessments of neural activity that may connect mechanistic research with clinically meaningful outcomes. The four contributions to this Research Topic illustrate complementary approaches toward this goal, from intracranial recordings during neuromodulation to non-invasive and multimodal measures in clinical and community samples.

## Electrophysiology as a bridge between mechanisms and clinical outcomes

Electroencephalography (EEG), event-related potentials (ERPs), local field potentials (LFPs), and analyses of neural oscillations can capture aspects of neural activity that are not readily inferred from symptom reports alone. This approach is consistent with the Research Domain Criteria framework, which encourages investigation of mental disorders across dimensions and levels of analysis, from circuits to behavior (1). In depression, neurobiological heterogeneity has also motivated efforts to identify subgroups with distinct patterns of brain connectivity and potentially different treatment responses (2). These findings support the rationale for biomarker research, while also underscoring the need for replication and independent validation before a marker is used to guide individual clinical decisions.

The clinical relevance of these measures is evident through circuit-based treatment that link physiology with clinical outcomes. Work on subcallosal cingulate deep brain stimulation (DBS) has used connectomic information to improve targeting of treatment-resistant depression (3). Meanwhile, functional near-infrared spectroscopy (fNIRS) offers a complementary measure of cortical hemodynamic responses and has been applied to several psychiatric conditions (4). Combining electrophysiological and hemodynamic measures may therefore help characterize different aspects of brain function, provided that the added complexity produces reliable and clinically useful information.

Preclinical studies complement clinical biomarker research by enabling neural activity to be examined at the level of individual neurons and local circuits during behavior. Recordings of single-unit activity and local field potentials, combined with targeted circuit manipulations, can help establish how neural signals contribute to specific cognitive processes and behavioral outcomes (5). Together, these approaches can help clarify the functional significance of electrophysiological markers identified in clinical populations.

## Contributions of this Research Topic

Ghazavi et al. examined the combinatorial effects of multi-site stimulation on depression-related brain regions using clinical data and predictive modeling, alongside intracranial LFP recordings during stimulation of the subcallosal cingulate and ventral capsule/ventral striatum. Their analyses suggest that interactions between stimulation sites and recorded signals are not uniform, with additive or sub-additive effects more common than super-additive effects. This work highlights the potential of intracranial physiology to inform stimulation strategies. However, the very small clinical sample means that the patterns and their relationship to therapeutic benefit require validation in larger cohorts.

Francis et al. investigated brain–gastric electrophysiology for early prediction of antidepressant response. By combining EEG, electrogastrography, and symptom profiles in a multicenter clinical study, the authors explored whether coordinated brain–body signals could help distinguish response patterns early in treatment. This multimodal approach is appealing because psychiatric symptoms occur in the context of broader physiological systems. Before clinical implementation, however, predictive performance and the generalizability of the model need to be confirmed in independent populations and under clearly specified clinical conditions.

Li et al. reported parallel electrophysiological and prefrontal hemodynamic alterations during Stroop task performance in major depressive disorder. Simultaneous EEG–fNIRS recordings provide complementary information about electrical activity and cortical hemodynamic responses during cognitive control. This design offers a broader view of task-related dysfunction than either modality alone. The reported group differences are informative, but further studies should establish their reproducibility, clarify how they relate to symptom dimensions and functional impairment, and determine whether they add value beyond behavioral performance or standard clinical assessment.

Mi et al. examined resting-state frontal theta/beta ratio and psychological symptoms in college students. In this sample, greater symptom burden was associated with differences in frontal theta/beta activity. The study adds research on accessible EEG indices in non-clinical populations and may help generate hypotheses about links between neural activity and psychological distress. These associations should not be interpreted as evidence that the ratio is a stand-alone diagnostic test or a validated predictor of future illness. Longitudinal work and assessment of specificity across symptom domains will be important in the next steps.

## Challenges and future directions

Taken together, these studies not only demonstrate the breadth of electrophysiological research but also point to shared priorities. First, reproducibility requires transparent reporting of acquisition protocols, preprocessing, feature selection, and statistical analyses. Where possible, researchers should assess test–retest reliability and use harmonized methods across sites. Second, identifying markers associated with neuropsychiatric symptoms does not necessarily establish their clinical relevance. Predictive studies should report appropriate measures of discrimination and calibration, compare models with relevant clinical baselines, and test performance in independent samples. Small datasets require particular care to prevent overfitting. Third, stronger links between preclinical mechanisms and human outcomes will require carefully designed translational studies. Candidate markers identified in experimental models should be assessed for comparable biological meaning in people, while recognizing differences between species, tasks, and recording conditions. Longitudinal studies can help establish whether a marker tracks change, predicts response, or merely correlates with symptoms at a single time point. Finally, multimodal approaches should be guided by a clear rationale: combining signals is useful only when it improves interpretability, reproducibility, or decision-making beyond simpler measures.

## Conclusion

The four articles in this Research Topic illustrate how electrophysiological and related physiological measures can connect neural mechanisms, symptom profiles, and treatment research. Their methods range from invasive recordings during DBS to non-invasive EEG and combined brain–body or EEG–fNIRS approaches. The next step is not simply to identify more candidate markers, but to establish which measures are reliable, reproducible, and useful for clinical decisions. Preclinical models can help guide this progress, but translating findings across species requires careful consideration of differences in behavior, underlying circuitry, and functional significant of electrophysiological activity. Progress will depend on larger and more diverse samples, independent validation, transparent methods, and close integration of mechanistic and clinical research. These improvements will help move the field beyond identifying neural correlates of neuropsychiatric symptoms toward understanding circuit mechanisms and identify novel targets for therapeutic intervention.
